# Admission Hemoglobin Associated with Increased Mortality in Hip Fracture Surgical Patients: An Observational Study

**DOI:** 10.3390/biomedicines12092041

**Published:** 2024-09-08

**Authors:** Ana Šarić Jadrijev, Ana Bego, Borna Lojpur, Dino Poljak, Marija Žaja, Jakov Matas, Božen Pivalica, Sanda Stojanović Stipić, Vesna Čapkun, Katarina Vukojević, Merica Glavina Durdov, Andre Bratanić

**Affiliations:** 1 Department of Anaesthesiology and Intensive Care, University Hospital of Split, Spinčićeva 1, 21000 Split, Croatia; ana.bega84@gmail.com (A.B.); marijazaja@hotmail.com (M.Ž.); sastojanovic@kbsplit.hr (S.S.S.); 2 Institute of Emergency Medicine in Split-Dalmatia County, Spinčićeva 1, 21000 Split, Croatia; borna.lojpur@gmail.com; 3 Department of Surgery, University Hospital of Split, Spinčićeva 1, 21000 Split, Croatia; dinopoljak@yahoo.com (D.P.); bozenpivalica@gmail.com (B.P.); 4 Priska Med Polyclinic, Kroz Smrdečac 45, 21000 Split, Croatia; jakovmatas@gmail.com; 5 Department of Anatomy, Histology and Embryology, School of Medicine, University of Split, Šoltanska 2A, 21000 Split, Croatia; vesna.capkun@gmail.com (V.Č.); kvukojev@gmail.com (K.V.); 6 Department of Pathology, Legal Medicine and Cytology, University Hospital of Split, Spinčićeva 1, 21000 Split, Croatia; merigdst@yahoo.co.uk; 7 Department of Internal Medicine, University Hospital of Split, Spinčićeva 1, 21000 Split, Croatia; andrebratanic@net.hr

**Keywords:** elderly, hip fractures, proximal femoral fractures, anemia, admission hemoglobin, transfusion trigger, transfusion, three time points, mortality

## Abstract

In hip fracture patients, who are mostly elderly, preexisting anemia can be worsened when combined with trauma and surgery. To this date, there is no unequivocal approach about transfusion thresholds. We analyzed hemoglobin (Hb) and hematocrit (Hct) levels at three time points in surgical patients with proximal femoral fractures (PFF) to see which levels were triggers for transfusions and whether transfusions were related to mortality after hospital discharge. A total of 956 patients were operated on from 1 January 2021 to 31 December 2022 at the University Hospital of Split and included in the study. There were more women (74%); 47% patients had admission Hb < 120 g/L. Transfusion was given preoperatively to 88, intraoperatively to 74 and postoperatively to 309 patients. Transfusion thresholds were as follows: Hb 84 g/L preoperatively, 99 intraoperatively and 83 postoperatively. After hospital discharge, 10.79% of patients died within the 1st month and 23% within 6 months. In the group of non-survivors, 60% of patients had admission Hb ≤ 117 g/L and the proportion of patients transfused preoperatively was two times higher. Preoperative transfusion thresholds could be set to higher levels for patients with surgically treated PFF. However, that could increase mortality even more. Further investigation is necessary.

## 1. Introduction

According to the World Health Organization (WHO), the aging of the human population is a global trend. Hip fractures are among the most common injuries and one of the leading causes of hospitalization in old and very old people [1,2]. They are usually the result of low-energy trauma and osteoporosis [3,4]. Hip fractures are actually fractures of the proximal part of the femur (PFF) that typically require surgical repair [5,6]. Patient mortality after these surgeries is relatively high, up to 36% within one year of injury, which is why perioperative care is a challenge for surgeons and anesthesiologists [7,8,9].

Patients with PFF very often have a complex medical background [10]. Many of them are anemic. Preexisting anemia can be aggravated by acute bleeding caused by trauma and subsequent surgery [5,7,11,12,13,14,15,16]. Even mild anemia before major surgical procedures increases the risk of morbidity and mortality [6,8,17,18]. The classic WHO criteria uses hemoglobin (Hb) values to define anemia [7]. Although Hb decreases with age, there is still no clear consensus about the appropriate Hb values for diagnosing anemia in the population of the elderly. Specifically, some authors consider anemia as when Hb is below 120 g/L in both sexes of elderly patients, while others consider it as when Hb is below 130 g/L [5,7,13,19]. Additionally, there are different types of anemia [11,20,21,22,23,24,25]. Anemia is often multifactorial and treatment options vary [7]. However, when Hb drops to a certain point and more than 40% of blood volume is acutely lost, a blood transfusion is absolutely required in order to enhance oxygen-carrying capacity and improve patients’ outcomes [26,27]. 

The Hb and hematocrit (Htc) values at which transfusion treatment should commence are known as transfusion thresholds. The liberal transfusion strategy (LTS) uses a Hb value of 100 g/L as a threshold for transfusion and aims to maintain Hb between 100 and 120 g/L [27]. However, transfusion services are based on voluntary donations and blood is an expensive, scarce resource [15,26,28]. What is more, blood transfusion is not without risks and should be considered as a transplantation [5,19,29]. That is why the Patient Blood Management (PBM) Guidelines support the restrictive transfusion strategy (RTS), which avoids exposition to heterologous blood transfusion [26,30,31]. According to the RTS, transfusion is indicated only when Hb drops < 70 g/L or symptoms of anemia are manifested in adult, non-bleeding critically ill patients [32,33]. In the perioperative setting and in the absence of acute myocardial or cerebrovascular ischaemia, transfusion is inappropriate for patients with Hb > 80 g/L and its value should be maintained between 70 and 90 g/L. Patients undergoing orthopedic or cardiac surgery and those with existing cardiovascular diseases have higher transfusion thresholds, a Hb value of 80 g/L [16,27]. Furthermore, some published papers concluded that no difference in mortality, functional recovery or postoperative morbidity was found between the two strategies for patients undergoing hip fracture surgery [5,28].

Different transfusion strategies have inspired us to conduct this research. The objectives of our study were to establish Hb and Htc values at three time points, particularly in relation to sex, to identify transfusion triggers and to determine if women were more likely to receive transfusions in our patient population. Furthermore, we aimed to investigate outcomes 1 and 6 months after hospital discharge and factors that had impacted our patients’ outcomes within a month of hospital discharge.

## 2. Materials and Methods

### 2.1. Patients and Study Design

This retrospective, single-center study was conducted in the University Hospital of Split, a tertiary care institution in Split, Croatia. Adult patients who were diagnosed with PFF in the Department of Surgery from 1 January 2021 to 31 December 2022 were our target population. Data were observed and collected in the first half of 2023, which makes our study observational and cross-sectional. Primary endpoints were the transfused volumes, number of doses transfused, Hb and Htc values at three time points and their differences related to erythrocyte concentrate transfusions (ECT). The secondary endpoint was mortality (in-hospital and 1 and 6 months within hospital discharge).

### 2.2. Inclusion and Exclusion Criteria

Only adults surgically treated in our institution were included in this study. That is, some of the patients who were our target audience were treated conservatively and some were foreigners that wanted to be treated in their respective countries. Polytraumatized patients with PFF were excluded from this study because their fractures were high-energy injuries. Foreigners who received surgical treatment in our institution were excluded as well because their follow-up was impossible. Additionally, a few patients were lost at follow-up.

### 2.3. Data Collection

Data collection was carried out retrospectively by analyzing patients’ medical records. We noted down their demographic and baseline characteristics, fractures that were the reason for hospitalization, type of surgeries and anesthesia, time to surgery (TTS), duration of stay in the intensive care unit (ICU), hospital length of stay (HLOS) and their outcomes after hospital discharge. When it comes to patients’ laboratory results, we decided to take into account Hb and Htc values at three time points; on admission to hospital, just before surgery (written on anesthesia records) and first postoperative values. Furthermore, anesthesia records were used to extract data concerning the physical condition of our patients. In particular, after evaluating their patients just before surgeries, anesthesiologists in our hospital used a physical status classification system created by the American Society of Anesthesiologists (ASA score) [33]. In addition to these values, we calculated the Charlson Comorbidity Index (CCI) and Elixhauser comorbidity index (ECI). We used free mobile application for calculating CCI, an MDCalc Medical Calculator, as well as a free online Elixhauser comorbidity index calculator and OrthoToolKit for ECI [34,35,36]. Both tests are based on the International Classification of Diseases 10th Revision (ICD-10). The CCI ranges from 0 to 29 and the ECI from −19 to 89. We stratified final results into 4 groups, for CCI: 0, 1–2, 3–4, ≥5, and for ECI: <0, 0, 1–4, ≥5 [37]. This stratification was found in the available literature and we accepted it because we wanted our results to be comparable to those published in similar articles. According to its authors, the CCI predicts the 10-year survival of patients and the 4 aforementioned groups are related to the percentage of survival. Zero points indicate that there are no comorbidities and predicted 10-year survival is 98%. Patients with 1 and 2 points have an estimated 10-year survival of 90 to 96%, patients with 3 and 4 points have a survival of 53 to 77% and patients with ≥5 points have an estimated survival equal or less than 21% [38]. Moreover, we formed the age groups as well, to evaluate more precisely the effect of age on mortality. TTS was defined as the time between hospital admission and surgery. Unfortunately, we did not have hours written in our patients’ medical records. That is why we expressed TTS in days. Data on mortality were obtained from medical records; however, the causes of deaths were not collected. Complications are not shown in this paper. 

### 2.4. Ethical Aspects

The study was conducted in accordance with ethical standards of the Declaration of Helsinki. Moreover, the Ethics Committee of University Hospital of Split gave approval to this study protocol (approval number: 500-03/22-01/21; date of approval: 3 March 2022). Informed consent was not necessary because the study was observational and retrospective.

### 2.5. Statistical Analysis

Statistical Package for the Social Sciences—SPSS 20.0 (IBM Corp, Armonk, NY, USA) and Microsoft Excel for Windows version 11.0 (Microsoft Corporation, Redmond, WA, USA) were used for statistical analysis. 

Medians and ranges (Q1–Q3; min–max) were used to describe the distribution of quantitative data, whereas categorical data were described with absolute numbers and percentages. For the comparison of continuous variables, the Mann–Whitney U test as well as the Wilcoxon test was applied. Categorical variables were compared using the chi-square (*Χ*^2^) test. All *p* values < 0.05 were considered significant. The binary logistic regression analysis was carried out as well as a calculation of the odds ratio (OR) of non-surviving/survived patients within a month of being discharged from the hospital, correlated with some important characteristics. Moreover, a receiver operating characteristic (ROC) analysis was performed to illustrate the performance of the non-surviving/survived model at varying threshold values for continuous variables. It was used to find the cut off value for continuous variables in order to separate the non-surviving and the survived patients within a month of hospital discharge [39].

## 3. Results

In a 2-year period, 1119 adults were diagnosed with PFF. A total of 1061 patients met inclusion criteria because they were adults treated surgically. Those who were polytraumatized and foreigners were excluded and some patients were lost at follow-up. That left us with 956 patients who were surgically treated due to PFF and had a complete follow-up after 6 months. 

This study included 246 (26%) male and 710 (74%) female patients. A total of 51% of women were in the ninth decade of their life. Further, 51% of patients were ASA 3, with 56% of patients scoring ≥ 5 points on the CCI and 47% on the ECI. A total of 67.5% patients had neither anticoagulants nor antiplatelet drugs in their therapy (Table 1). The proportion of patients younger than 61 was 5.1 times higher in the group of men than in the group of women. The proportion of patients aged between 72 and 81, 82 and 91 and ≥92 years was 1.32, 1.28 and 1.44 times higher in the group of women than in the group of men. This difference was statistically significant (*Χ*^2^ = 69.1; *p* < 0.001). The proportion of ASA 1 patients in the group of men was 8.1 times higher than in the group of women. The proportion of patients with 0 points according to CCI was 10 times higher in the group of men than in the group of women. The proportion of patients with an ECI score < 0 was 2.1 times higher in the group of men than in the group of women. The distributions of patients according to ASA classification, the CCI and the ECI were statistically significantly different in relation to sex (*Χ*^2^ = 29.9; *p* < 0.001, *Χ*^2^ = 37.8; *p* < 0.001 and *Χ*^2^ = 9.3; *p* = 0.025, respectively). 

Our patients were 1.5 times more often diagnosed with extracapsular than with intracapsular fractures (58.6% and 39.3%, respectively) (Table 2). Femoral neck fractures are intracapsular as they occur within the capsule of the hip joint. Extracapsular fractures, intertrochanteric and subtrochanteric, have a fracture line somewhere between the greater and lesser trochanter or up to 5 cm below the lesser trochanter [1]. We did not find a statistically significant difference between men and women according to the type of fracture (*Χ*^2^ = 2.78; *p* = 0.428). The most frequently performed surgery was proximal femoral nail (PFN). We did not find a statistically significant difference between men and women according to the type of surgery (*Χ*^2^ = 1.26; *p* = 9.739). A total of 85% patients were operated under spinal anesthesia. We did not find a statistically significant difference between men and women according to the type of anesthesia (*Χ*^2^ = 0.491; *p* = 0.484). The medians for TTS, ICU stay and HLOS were 3, 0 and 10 days, respectively (Table 3). For 763 (82.4%) patients, the discharge location was their own home. 

Medians of Hb at admission, on anesthesia records and the first postoperative ones, were 121, 120 and 103.5 g/L, respectively. Medians of Hct at admission, on anesthesia records and first postoperative ones, were 0.36, 0.36 and 0.31 L/L, respectively. Medians of Hb were 8, 7 and 6 g/L higher in men than in women (Table 4). Medians of Htc were 0.02 higher in men than in women. A total of 451 (47%) patients were anemic at admission. Moreover, 359 (79.6%) female and 92 (20.4%) male patients had Hb < 120 g/L at admission and this difference was statistically significant (*Χ*^2^ = 12; *p* = 0.001).

### 3.1. Hb and Htc Differences Related to ECT

A total of 88 (9.2%) patients received transfusion preoperatively, 74 (7.7%) intraoperatively and 309 (32.3%) patients postoperatively (Table 5). There were no differences between men and women when it comes to the number of transfused doses and total volumes of erythrocytes transfused preoperatively (*Χ*^2^ = 0.123; *p* = 0.888 and Z = 0.566; *p* = 0.571), intraoperatively (*Χ*^2^ = 3.36; *p* = 0.187 and Z = 1.45; *p* = 0.147) and postoperatively (*Χ*^2^ = 3.36; *p* = 0.187 and Z = 0.566; *p* = 0.571). 

Preoperative thresholds for transfusion for men and women were 85 g/L and 83 g/L, intraoperative 111 g/L and 96 g/L and postoperative 85 g/L and 83 g/L, respectively (Table 6). After preoperative transfusion, the median value of Hb (Z = 6.8; *p* < 0.001) as well as the median value of Htc (Z = 6.7; *p* < 0.001) increased. Values of Hb increased in 72 (89%) patients who received preoperative transfusion. Hb increased in 15 (88%) men and in 57 (89%) women (*Χ*^2^ = 0.01; *p* = 0.921). Values of Htc increased in 69 (85%) patients who received preoperative transfusion. Htc increased in 13 (76%) men and in 56 (87%) women (*Χ*^2^ = 1.3; *p* = 0.258). There was no statistically significant difference between values of Hb (Z = 0.073; *p* = 0.942) and Htc (Z = 0.426; *p* = 0.670) after intraoperative transfusions. Values of Hb increased in 34 (47%) patients who received intraoperative transfusions. Hb increased in four (21%) men and 30 (55%) women (*Χ*^2^ = 6.7; *p* = 0.010). Values of Htc increased in 31 (42%) patients who received intraoperative transfusions. Htc increased only in two (11%) men and 29 (53%) women (*Χ*^2^ = 10.6; *p* = 0.001). The proportion of increased Hb was 2 times higher in women than in men. The proportion of increased Htc was 4.8 times higher in women than in men. After postoperative transfusion, the median value of Hb increased (Z = 13.9; *p* < 0.001) as well as the median value of Htc (Z = 13.5; *p* < 0.001). Values of Hb increased in 278 (92%) patients who received postoperative transfusion. Hb increased in 71 (89%) men and in 206 (93%) women (*Χ*^2^ = 1.6; *p* = 0.207). Values of Htc increased in 268 (89%) patients who received postoperative transfusion. Hb increased in 66 (82%) men and in 201 (91%) women (*Χ*^2^ = 3.6; *p* = 0.066). 

### 3.2. Follow-Up

We identified 14 patients, 7 (17%) males and 7 (12%) females, who died before hospital discharge. A total of 100 (10.8%) patients died within 1 month and 207 (23.4%) within 6 months of hospital discharge (Table 7). The proportion of non-survivors was 2.2 times higher in the group of men than in the group of women who died within a month of hospital discharge (*Χ*^2^ = 14.9; *p* < 0.001). The proportion of non-survivors was 1.5 times higher in the group of men than in the group of women who died within 6 months of hospital discharge (*Χ*^2^ = 9.9; *p* = 0.002). 

In the group of non-survivors who died within 1 month of hospital discharge (n = 100), preoperative transfusions resulted in Hb and Htc increases in both men and women. A total of seven men in the group of non-survivors received preoperative transfusion, six (85%) experienced Hb increase and five (71%) experienced Htc increase. A total of eight women in the group of non-survivors received preoperative transfusion; all of them (100%) experienced a Hb increase and seven (87%) experienced a Htc increase. In the group of non-survivors who died within 1 month of hospital discharge, 14 men and 21 women received intraoperative transfusions and that led to a decrease in Hb and Htc levels in both men and women. A total of 13 (93%) men experienced Hb decrease and in 11 (79%) of them Htc decreased. A total of 19 (90%) women experienced both Hb and Htc decreases. In the group of non-survivors who died within 1 month, 16 men and 21 women received transfusions postoperatively and in both sexes transfusions resulted in Hb and Htc decreases. Moreover, seven (43%) men experienced Hb and Htc decreases and in nine (56%) of them Hb and Htc stayed the same. In 17 (81%) women, both Hb and Htc stayed the same. 

The proportion of men in the group of non-survivors was 1.7 times higher than the proportion of men in the group of survivors (*Χ*^2^ = 14.9; *p* < 0.001) (Table 8). The odds that death would occur were 2.3 times higher in the group of men than in the group of women (*p* < 0.001). The distribution of patients according to age groups was statistically significantly different in relation to fatal outcomes. The proportion of patients between 82 and 91 years old was 1.4 times higher in the group of non-survivors than in the group of survivors. The odds of death increased by 2.1 times with each increase in age groups (*p* < 0.001). The distribution according to the ASA classification was statistically significantly different in relation to the outcome for those who died within a month of hospital discharge. The proportion of patients with ASA 3 in the group of non-survivors was 1.4 times higher than in the group of survivors (*Χ*^2^ = 34; *p* < 0.001). The odds of death increased with each category of ASA score by 2.9 times (*p* < 0.001). The proportion of patients with CCI ≥ 5 was 1.4 times higher in the group of non-survivors than in the group of survivors (*Χ*^2^ = 23, *p* < 0.001). The odds of death increased with each CCI category by 2.4 times (*p* < 0.001). The proportion of patients with ECI ≥ 5 was 1.44 times higher in the group of non-survivors than in the group of survivors (*Χ*^2^ = 15.5; *p* = 0.001). The odds of death increased with each category of ECI score (*p* = 0.001). ROC analysis (area: 0.607; SE 0.030; 95% CI: 0.55–0.666; *p* < 0.001) gave us the cut-off value of admission Hb in relation to fatal outcomes. In the group of non-survivors (n = 100), 60 patients had a Hb ≤ 117 g/L, so the sensitivity was 60%. In the group of survivors (n = 825), 483 patients had a Hb > 117, so the specificity was 58% (*Χ*^2^ = 12.5; *p* < 0.001). The odds of death were 2.1 times higher in the group of patients who had a Hb ≤ 117 g/L compared to the group of patients who had a Hb > 117 g/L (*p* = 0.001). The proportion of patients who were transfused preoperatively was 2 times higher in the group of non-survivors than in the group of survivors (*Χ*^2^ = 7.8; *p* = 0.005). The odds of death were 2.3 times higher in the group of those who received preoperative transfusion (*p* = 0.004). We proved a statistically significant association between neither intraoperative (*Χ*^2^ = 0.084; *p* = 0.772) nor postoperative (*Χ*^2^ = 1.84; *p* = 0.175) transfusions with fatal outcomes. The proportion of patients who were operated on after 2 days was 1.36 times higher in the group of non-survivors than in the group of survivors (*Χ*^2^ = 12.4; *p* < 0.001). The odds of death in the group of patients operated on after 2 days were 2.3 times higher than in the group of patients who were operated on within 2 days (*p* < 0.001). The proportion of patients operated on under general anesthesia was 1.7 times higher in the group of non-survivors than in the group of survivors (*Χ*^2^ = 6.1; *p* = 0.014). The odds of death in patients operated under general anesthesia were 2 times higher than in patients operated under spinal anesthesia (*p* = 0.010).

The distribution of patients according to those who took anticlotting agents before hospitalization (*Χ*^2^ = 1.44; *p* = 0.838) and the type of fracture (*Χ*^2^ = 0.588; *p* = 0.899) did not differ statistically significantly in relation to fatal outcome within a month of hospital discharge.

## 4. Discussion

This study was designed with an intention to improve perioperative care in our institution and is getting us one step closer to creating a standardized protocol for the perioperative care of surgical patients with PFF. The aims of this study were to establish Hb and Htc values at three time points, particularly in relation to sex, to identify Hb and Htc values that triggered transfusions and to determine if women received more transfusions. Furthermore, this study aimed to investigate outcomes 1 and 6 months after hospital discharge and factors that affected the mortality of our patients within a month of hospital discharge. Here, there are two sides of a coin when it comes to transfusions and their possibility to affect outcomes. Transfusions are associated with the development of acute kidney injury, increased mortality and increased risk of infection [40,41]. The most common reason for death related to transfusing blood products is transfusion-associated circulatory overload [19]. On the other hand, transfusions do improve results when it comes to hospitalizations and mortality and adequate patient blood management improves outcomes of surgical patients with PFF [27]. 

The most interesting finding of our study was that the group of patients with Hb ≤ 117 g/L at admission to hospital had 2.1 times higher odds of death compared to the group of patients who had admission Hb > 117 g/L. According to many papers, low preoperative Hb is a significant predictor of mortality after hospital discharge [6,42]. The existing literature documents that initial Hb between 80 g/L and 100 g/L is a strong predictor of death [43,44]. A similar study indicates the association between initial Hb and mortality up to 3 years post-surgery, with a Hb of <98 g/L as an inflection point. Nevertheless, our Hb cut off value was considerably higher. Investigators from Denmark published similar results to ours. Their finding was that Hb at admission as low as 120 g/L was an independently associated factor of early and one-year mortality in elderly, fragile hip fracture patients, together with the need for transfusion, an age greater than 80 years and the male sex [8]. 

In our group of patients, Hb and Htc were higher in men than in women at three time points and those differences were statistically significant. That is, in accordance with data that men and women normally have different mean Hb and Htc values and that mean levels in women are approximately 12% lower than in men [45]. Correspondingly, our institution’s laboratory has different Hb and Htc reference values for men (138–175 g/L and 0.415–0.530 L/L, respectively) and women (119–157 g/L and 0.356–0.470 L/L, respectively).

Anemia is usually common in the preoperative setting, affecting around 1/3 of patients programmed for major surgery [18]. Preoperative anemia is frequent at the hospital admission of elderly patients with hip fractures, which makes it a hot topic when it comes to surgical PFF patients [8]. 

Our anemia threshold was set at 120 g/L for both sexes. When it comes to anemia in our patient population, a lot of our patients had Hb < 120 g/L at admission to hospital, a total of 47% of them. If we had used redefined criteria, the percentage would be even higher. That is, since the anemia threshold was redefined at 130 g/L for both sexes, its prevalence rose even higher [18]. Our numbers are in accordance with available papers. In the available literature, the incidence of anemia varies from 12.3% to 40.4%, depending on the Hb value set as a threshold for diagnosing anemia [8]. The prevalence of anemia before operation has been found to be as high as 60% [46]. Additionally, one paper reported the prevalence of preoperative anemia as 44 ± 9% and that postoperative anemia was even higher, 87 ± 10% [47]. These high values and the fact that the prevalence of anemia ≥ 40% has severe public health significance cannot be ignored, especially regarding preoperative anemia [25]. Treatment of the cause of anemia is preferable to transfusion, meaning that transfusion is not the only treatment option. It should only be offered for the treatment of symptomatic anemia and for the replacement of traumatic or surgical blood loss. There are three pillars of care according to PBM: the optimization of red cell mass and erythropoiesis before operation, the minimization of blood loss intraoperatively and the management of postoperative anemia [31]. However, PFF surgeries are urgent and there is not too much time for patients’ optimization. Postponing surgeries could increase the time between hospital admission and surgery (or the time between fracture itself and surgery) which is an important factor if trying to decrease HLOS and mortality [48]. 

The median TTS in our institution was 3 days, for both men and women. The standard for surgical fixation of hip fractures is 48 h [48]. There is a dispute in the literature regarding the impact of surgical timing on outcomes [44]. Some studies challenge the unwritten rule that PFF surgery needs to be performed within 2 days of hospital admission because they do not find a connection between delaying surgery and unwanted outcomes [49,50,51]. They have found that delaying surgery for up to 4 days was not connected to higher morbidity or mortality and concluded that preoperative optimization of patients’ conditions was far more important than being bound to a universal timing of surgery. Nevertheless, the majority of studies concluded that surgical fixation beyond 48 h after hip fracture resulted in an increased HLOS and 1-year mortality [48]. There are even some papers discussing the effect of accelerated surgeries on mortality. Their median time from diagnosis to surgery was 6 h but they did not find a significantly lower risk of mortality (when compared to the standard of care which, in their research, was 24 h from the diagnosis) [52]. In our patient population, TTS had a significant effect on mortality. Patients operated on after 2 days in our institution had 2.3 times higher odds of death than those operated in less than 2 days from diagnosis. 

Preoperative thresholds for transfusion for men and women were 85 g/L and 83 g/L, intraoperative 111 g/L and 96 g/L and postoperative 85 g/L and 83 g/L, respectively. That means that we were closer to the RTS in our daily practice. That was somehow expected because PBM was gaining popularity over the years and the RTS was proven to be safe both for patients admitted to the intensive care units and those undergoing surgery. However, our thresholds were higher intraoperatively because of clinicians’ estimates, which made us closer to the LTS. The LTS does not increase the risk of infection in transfused patients and improves survival [40,53,54]. What is more, we did not have point of care devices near our operating theaters and the decision of when to transfuse was made after taking into account the amount of blood in the aspirator and discussing the particular patient with the surgeons. Since anesthesia records did not have data on patients’ weight and height, we can only speculate on whether anesthesiologists calculated allowable blood losses or not.

Despite a high percentage of anemia at hospital admission, most transfusions occurred postoperatively. This is similar to findings described in the literature [29,41,55]. When it comes to transfusions and mortality, we found an association between neither intraoperative nor postoperative transfusion and mortality 1 month after hospital discharge. Only patients who received preoperative transfusions had higher odds of death. Some authors found no association between transfusions and the overall survival of elderly patients undergoing hip fracture surgery; others did, especially with intraoperative transfusion [8,41,56,57]. 

Our results showed that there was no statistically significant difference in the number of doses and volumes transfused between men and women at the three time points. Additionally, we used the same transfusion thresholds for both sexes. If we bear in mind that women normally have lower Hb levels, this actually means that we unintentionally transfused women more. Further research is necessary to determine if this was the reason why the women in our study had better outcomes within a month of being discharged from hospital.

The Hb and Htc differences showed a good response to ECT in both men and women preoperatively and postoperatively. The differences correlated positively with the given number of doses and total volumes. Intraoperative transfusion did not show the same effect: there was a significant difference between sexes. There were decreases in men and increases in women. Moreover, we found no significant correlation between Hb and Htc differences and neither the number of doses nor the total volumes transfused. Further research is needed to enlighten this observation. There are probably additional factors which need to be taken into account (the amount of intraoperatively administered crystalloids, surgeons operating, anesthesiological and surgical techniques, etc.).

Regarding fatal outcomes, within a month of hospital discharge, a total of 100 patients died, resulting in a one-month post-discharge mortality rate of 10.79%. Within 6 months of hospital discharge, 207 patients died, leading to a 6-month post-discharge mortality rate of 23%. According to the literature, mortality rates for patients undergoing surgery for PFF vary: 8% in the United Kingdom, 11.2% after 3 months of follow-up and 14.1% after 6 months, and when followed up after a longer period up to 35% [6,9,58]. That implies that fatal outcomes are not necessarily related to the surgery itself [59,60]. On the other hand, high mortality rates are somehow expected, since these patients are often elderly, have multiple comorbidities and are on numerous medications [2,44,61,62,63].

If we focus on baseline characteristics, when it comes to sex, women predominated in our research. They accounted for 74% of our patients, which means that women suffered PFF 2.9 times more often than men. The same proportion can be found in the literature but this percentage can be even higher, up to 79% [41,55,64,65,66].

The majority of patients (59%) operated on in our institution were older than 82 years old. Similar reports are seen in the available literature [65,66]. In our study, the odds of death within a month of hospital discharge increased by 2.1 times with each age group. In our patient population, the male sex was more common in younger age groups and the female sex was more common in older age groups. This aligns with the United Nations World Social Report 2023 stating that women live an average of 5.4 years longer than men, comprising 56% of the population over 65 and 62% of the population over 80 years old. Although there are predictions that by 2050 men’s survival rates will approach those of women, women will probably still outnumber men over 65 and 80 years old, though the balance between men and women will be more equal. In that light, in our study, the odds of death within a month of being discharged from hospital were 2.3 times higher in men than in women.

The majority of PFF patients operated on in our institution were seriously ill, just like those described in the literature [62,67]. These patients face a significant risk of death due to their comorbidities, as well as advanced age, frailty and polypharmacy. Because comorbidity status can predict perioperative death, guidelines for the perioperative care of patients with PFF recommend using risk assessment tests [67,68,69,70,71]. It remains unclear how much new trauma, surgery and anesthesia contribute to the overall risk of mortality and perioperative complications. The experience of anesthesiologists plays a critical role in assessing these risks, explaining these to patients and their caregivers and coordinating with other clinicians [69]. Various risk assessment tests are available in the literature but, to this date, an optimal score for orthopedic patients does not exist. In our institution, the ASA physical status classification system is routinely used to evaluate comorbidities. According to the American Association of Anesthesiologists, ASA score alone is not ideal for assessing perioperative risks, but it can be useful when combined with other factors. Some authors agree with this claim, saying that CCI and ECI are better than ASA classification in predicting one-year mortality after PFF [67]. Others state that ASA score is reliable in predicting postoperative outcomes [72]. For this reason, we determined ASA scores as well as the CCI and ECI for each individual patient who entered our study. When it comes to the CCI, the higher the score, the greater the likelihood of increased mortality or higher resource utilization [73]. The ECI is considered to be more effective than the CCI in predicting outcomes [34,36,37]. As previously mentioned, our male patients were younger, but also healthier according to the ASA, CCI and ECI. The odds of death within a month of hospital discharge increased with each category of ASA score, CCI and ECI.

Anticoagulants are often used in patients with PFF. It is known that using these medications prolongs TTS and they are hence potentially associated with worse outcomes [74,75,76,77]. In one paper, nearly 50% of patients were on anticoagulants or anti-platelet therapy [78]. According to novel guidelines, 30–40% of people with PFF in the UK are taking some anticoagulant/antiplatelet medications and 2% are taking NOAC [69]. This is similar to our population: 32.5% of our patients used some anticoagulant/antiplatelet medications. Additionally, 11.8% of our patients were on some form of NOAC when admitted to the hospital. 

In the available literature, women more frequently experience intertrochanteric fractures [2,66]. This variation is explained with differences in bone density and the nature of falls between men and women [65]. However, in our patient population, we found a statistically significant difference neither between the type of fracture nor between the type to surgery and sex. Moreover, we did not find a statistically significant difference between non-survivors and survivors within a month after being discharged from hospital when it comes to the type of fracture. 

A total of 85% patients were operated on under spinal anesthesia. We are proud of that percentage because it has been proven that a central neuraxial blockade significantly reduces perioperative blood loss. Spinal anesthesia is considered as a blood-sparing strategy [31,79]. However, debate over the optimal type of anesthesia for hip fracture surgeries is ongoing [80,81]. Keeping that in mind, we found a statistically significant difference between non-survivors and survivors within a month of hospital discharge when it comes to the type of anesthesia. The odds of death were higher in patients operated under general anesthesia. Further research is needed to clarify if perioperative blood loss is the reason for those results.

This study is not without limitations. Firstly, it is of observational origin. It would be better if we could have conducted randomized controlled trials to better explain the role of admission Hb and other clinical parameters as predictors of adverse outcomes. However, that was not possible for us. PFF surgeries were urgent, a lot of different anesthesiologists and surgeons were involved and we did not have the ability to influence them, particularly because our institution does not have, to this date, standardized protocols for the perioperative treatment of PFF surgical patients. The second limitation is a lack of certain data. Some are lacking because they were not included in patients’ medical records (weight and height). That is why we were not able to calculate body mass indexes and total blood losses. Others are lacking because we decided not to include them in this study. We did not take into account CRP, urea, creatinine, eGFR, Fe and ferritin, even though we were aware that omitting them could limit our work. Inflammation and renal diseases impact Hb and Hct values in the population of the elderly [20,21,22,24]. There is an increase in anemia in the population of the elderly and in one third of these patients anemia is due to nutritional deficiency, including of iron [7]. However, at the time of data collection, we thought all these patients and operations were urgent. Low Hb and Hct were consequences of their preexisting anemia, combined with trauma and surgery, and transfusion was the only option for optimizing them in a short period of time. Diagnoses included in the scores on our patients’ medical status are also lacking because we did not write them down separately. We simply did not have the funds to put everything down and conduct such a study.

## 5. Conclusions

Lower transfusion thresholds, according to the RTS, were used when taking care of patients with PFF before and after surgery. Our intraoperative thresholds, however, were higher because decisions on whether to transfuse were made only on the basis of clinical parameters. Our results showed that an equal number of doses and equal volumes were transfused to men and women, which means that we actually transfused women more. Further research is needed to explain if that was the reason for the better outcomes of women within a month of hospital discharge. Higher odds of fatal outcome within 1 month of discharge were associated with the male sex, older age, more comorbidities, admission Hb ≤ 117 g/L, preoperative transfusions, general anesthesia and performing the operation ≥ 2 days after the diagnosis. 

To conclude, even though the PBM concept stresses the importance of the RTS in order to decrease the number of people exposed to transfusions, our results and admission Hb levels speak in favor of the LTS. We believe that this study, along with previously published research with similar results, should be considered relevant for this group of patients. Nevertheless, further research is needed to clarify if preoperative transfusion thresholds should be increased and how that would impact clinical outcomes and mortality. Other methods for patients’ optimization, that do not delay surgery, should be considered.

## Figures and Tables

**Table 1:** Demographic and baseline characteristics between men and women.

	Men (n = 246)	Women (n = 710)	*p* *
	n (%)		
Age groups (years)			<0.001
≤61	44 (17.9)	25 (3.5)	
62–71	38 (15.4)	64 (9)	
72–81	46 (18.7)	176 (24.8)	
82–91	98 (39.8)	362 (51)	
≥92	20 (8.1)	83 (11.7)	
ASA score			<0.001
ASA 1	14 (5.7)	5 (0.7)	
ASA 2	98 (39.8)	322 (45.4)	
ASA 3	121 (49.2)	367 (51.7)	
ASA 4	13 (5.3)	16 (2.3)	
CCI			<0.001
CCI 0	8 (3.3)	2 (0.3)	
CCI 1–2	43 (17.5)	55 (7.7)	
CCI 3–4	81(32.9)	232 (32.7)	
CCI ≥ 5	114 (46.3)	421 (59.3)	
ECI			0.025
ECI < 0	15 (6.2)	20 (2.9)	
ECI 0	78 (32.4)	189 (27.2)	
ECI 1–4	43 (17.8)	154 (22.1)	
ECI ≥ 5	105 (43.6)	333 (47.8)	
Anticlotting drugs			0.823
No	154 (68.8)	426 (67.1)	
Aspirin	29 (12.9)	93 (14.6)	
NOAC	32 (14.3)	72 (11.3)	
Warfarin	9 (4)	32 (5)	
LMWH	0 (0)	1 (0.2)	
Clopidogrel	0 (0)	3 (0.5)	

* *Χ*^2^ test. Abbreviations: ASA score—American Society of Anesthesiologists physical status classification system; CCI—Charlson comorbidity index; ECI—Elixhauser comorbidity index; NOAC—non-vitamin K antagonist oral anticoagulants; LMWH—low-molecular-weight-heparin.

**Table 2:** Surgical characteristics, type of anesthesia and time to surgery between men and women.

	Men (n = 246)	Women (n = 710)	*p* *
	n (%)		
Type of fracture			0.428
Femoral neck	102 (41.5)	273 (38.6)	
Intertrochanteric	117 (47.6)	361 (51.1)	
Subtrochanteric	24 (9.8)	56 (7.9)	
Periprosthetic	3 (1.2)	17 (2.4)	
Type of surgery			0.739
PFN	137 (56.1)	399 (56.4)	
PEP, BEP	71 (29.1)	204 (28.9)	
TEP	14 (5.9)	30 (4.2)	
Osteosynthesis, Revision	22 (9)	74 (10.5)	
Type of anesthesia			0.485
GA	33 (17)	85 (14)	
SA	164 (83)	507 (86)	
TTS (days)			0.855
<2	111 (46)	128 (54)	
≥2	317 (45)	389 (55)	

* *Χ*^2^ test. Abbreviations: PFN—proximal femoral nail; PEP—partial endoprothesis; BEP—bipolar endoprothesis; TEP—total endoprothesis; GA—general anesthesia; SA—spinal anesthesia; TTS—time to surgery.

**Table 3:** Time to surgery and lengths of stay (expressed in days) between men and women.

	Men (n = 246)	Women (n = 710)	*p* ^†^
	Median (Q1–Q3; Min–Max)		
TTS	3 (1–5; 0–27)	3 (1–4; 0–16)	0.879
ICU stay	0 (0–1; 0–20)	0 (0–1; 0–22)	0.085
HLOS	10 (8–13; 0–35)	10 (8–12; 0–50)	0.906

^†^ Mann–Whitney test. Abbreviations: ICU—intensive care unit; HLOS—hospital length of stay.

**Table 4:** Hemoglobin and hematocrit values at three time points between men and women.

	Men (n = 246)	Women (n = 710)	*p* ^†^
	Median (Q1–Q3; Min–Max)		
Hb (g/L)			
admission	127 (111–140; 64–180)	119 (105–129; 56–167)	<0.001
anesthesia record	126 (111–137; 76–180)	119 (106–129; 59–161)	<0.001
first postoperative record	108 (95–120; 59–172)	102 (91–113; 59–154)	<0.001
Htc (L/L)			
admission	0.37 (0.33–0.41; 0.2–0.6)	0.35 (0.32–0.38; 0.16–0.8)	<0.001
anesthesia record	0.37 (0.33–0.41; 0.2–0.6)	0.35 (0.32–0.38; 0.18–0.5)	<0.001
first postoperative record	0.32 (0.28–0.35; 0.2–0.5)	0.3 (0.27–0.34; 0.18–0.5)	0.001

^†^ Mann–Whitney test. Abbreviations: Q1—the first quartile; Q3—the third quartile; min—the minimum value; max—the maximum value; Hb—hemoglobin; Htc—hematocrit.

**Table 5:** Total volume and number of doses transfused at three time points between men and women.

Preoperatively:	Men (n = 18)	Women (n = 70)	*p*
Number of doses, n (%)			0.888 *
1	8 (47.1)	28 (40.6)	
2	6 (35.3)	27 (39.1)	
>2	3 (17.6)	14 (20.3)	
Volume (mL)	510 (255–530; 230–1030)	495 (262–537; 240–1330)	0.571 ^†^
Intraoperatively:	Men (n = 20)	Women (n = 54)	*p*
Number of doses, n (%)			0.187 *
1	10 (50)	36 (66.7)	
2	8 (40)	17 (31.5)	
>2	2 (10)	1 (1.9)	
Volume (mL)	520 (340–722; 280–790)	270 (245–490; 200–530)	0.147 ^†^
Postoperatively:	Men (n = 80)	Women (n = 229)	*p*
Number of doses, n (%)			0.187 *
1	22 (27.5)	67 (29.2)	
2	28 (35)	122 (53.3)	
>2	30 (37.5)	40 (17.4)	
Volume (mL)	520 (270–780; 230–1820)	510 (277–540; 210–1530)	0.039 ^†^

* *Χ*^2^ test. ^†^ Mann–Whitney test; volume was expressed as median (Q1–Q3; min–max).

**Table 6:** Transfusion thresholds and differences between Hb and Hct at three time points between men and women.

		Before ECT	After ECT	Difference	*p* ^‡^
Preoperatively					
Total n = 88	Hb	84 (73–89; 56–153)	97 (92–103; 78–132)	15 (6.5–24; −22 to 49)	<0.001
	Hct	0.25 (0.2–0.3; 0.2–0.4)	0.29 (0.28–0.31; 0.25–0.4)	0.04 (0.02–0.06; −0.07 to 0.15)	<0.001
Men n = 18	Hb	85 (82–92; 64–158)	97 (92–104; 89–132)	12 (4.5–22; −21 to 35)	0.010
	Hct	0.255 (0.25–0.3; 0.15–0.45)	0.29 (0.28–0.81; 0.26–0.38)	0.04 (0–0.06; −0.07 to 0.10)	0.020
Women n = 70	Hb	83 (71–88; 56–123)	96.5 (92–103; 78–125)	15 (8–26; −22 to 49)	<0.001
	Hct	0.26 (0.2–0.3; 0.2–0.4)	0.29 (0.28–0.31; 0.25–0.58)	0.04 (0.02–0.06; −0.04 to 0.15)	<0.001
Intraoperatively					
Total n = 74	Hb	99 (91–115; 59–152)	101 (94–112; 66–138)	−0.50 (−8.5 to 9; −0.45 to 49)	0.942
	Hct	0.30 (0.3–0.35; 0.2–0.4)	0.305 (0.3–0.33; 0.2–0.40)	0 (−0.03 to 0.03; −0.17 to 0.13)	0.670
Men n = 20	Hb	111 (94–124; 76–152)	101 (87–112; 66–138)	−6 (−15 to 0; −45 to 25)	0.035
	Hct	0.35 (0.3–0.4; 0.2–0.4)	0.29 (0.3–0.33; 0.2–0.4)	−0.02 (−0.06 to 0; −0.17 to 0.07)	0.025
Women n = 54	Hb	96 (89–113; 59–138)	101 (95–110; 79–137)	3 (−6 to 10; −32 to 49)	0.190
	Hct	0.29 (0.3–0.34; 0.2–0.4)	0.305 (0.29–0.33; 0.23–0.39)	0.01 (−0.02 to 0.03; −0.10 to 0.13)	0.323
Postoperatively					
Total n = 309	Hb	83 (78–88; 57–129)	99 (93–105; 80–126)	16 (8 to 24; −26 to 57)	<0.001
	Hct	0.25 (0.2–0.3; 0.2–0.4)	0.29 (0.26–0.29; 0.22–0.39)	0.04 (0.02–0.07; −0.08 to 0.18)	<0.001
Men n = 80	Hb	85 (78–92; 57–129)	99 (94–105; 80–126)	13 (5–21; −25 to 55)	<0.001
	Hct	0.25 (0.23–0.27; 0.2–0.4)	0.29 (0.28–0.31; 0.23–0.39)	0.04 (0.02–0.07; −0.08 to 0.18)	<0.001
Women n = 229	Hb	83 (77–87; 59–117)	99 (92–105; 81–123)	16 (9–24; −26 to 57)	<0.001
	Hct	0.25 (0.23–0.26; 0.2–0.3)	0.29 (0.3–0.31; 0.2–0.4)	0.05 (0.03–0.07; −0.08 to 0.26)	<0.001

^‡^ Wilcoxon test; all variables were expressed as median (Q1–Q3; min–max). Hb was expressed as g/L; Htc as L/L.

**Table 7:** Mortality after hospital discharge between men and women.

	Men	Women	*p* *
	n (%)		
Within 1 month			<0.001
Non-survivors	42 (18)	58 (8)	
Survivors	195 (82)	631 (92)	
Within 6 months			0.002
Non-survivors	71 (31)	136 (21)	
Survivors	156 (69)	520 (79)	

* *Χ*^2^ test.

**Table 8:** Characteristics of non-survivors and survivors within a month of hospital discharge.

	Non-Survivors	Survivors	*p* *	OR (95% CI)	*p* ^∮^
	n (%)				
Sex			<0.001	2.3 (1.5–3.6)	<0.001
Men	42 (42)	195 (24)			
Women ^‖^	58 (58)	631 (76)			
Age groups			<0.001	2.1 (1.6–2.7)	<0.001
≤61 ^‖^	1 (1)	67 (8.1)			
62–71	3 (3)	96 (11.6)			
72–81	13 (13)	205 (24.8)			
81–91	63 (63)	382 (46.2)			
≥92	20 (20)	77 (9.3)			
ASA score			<0.001	2.9 (2–2.4)	<0.001
1 ^‖^	0 (0)	19 (2.3)			
2	23 (23)	382 (46.2)			
3	67 (67)	407 (49.2)			
4	10 (10)	19 (2.3)			
CCI			<0.001	2.4 (1.6–3.6)	<0.001
0 ^‖^	1 (1)	9 (1.1)			
1–2	2 (2)	94 (11.4)			
3–4	19 (19)	280 (33.9)			
≥5	78 (78)	444 (53.7)			
ECI			<0.001	1.5 (1.2–2)	0.001
<0 ^‖^	4 (4)	32 (3.9)			
0	15 (15.2)	244 (29.9)			
1–4	16 (16.2)	175 (21.5)			
≥5	64 (64.6)	364 (64.7)			
Admission Hb			<0.001	2.1 (1.4–3.2)	0.001
≤117	60 (60)	342 (41)			
>117 ^‖^	40 (40)	483 (58)			
Preop ECT			0.005	2.3 (2.4–4.2)	0.004
No ^‖^	83 (83)	761 (92)			
Yes	17 (17)	86 (8)			
TTS (days)			<0.001	2.3 (1.4–3.6)	< 0.001
<2 ^‖^	28 (28)	389 (47)			
≥2	72 (72)	438 (53)			
Anesthesia			0.014	2 (1.2–3.5)	0.01
GA	21 (24)	98 (14)			
SA ^‖^	65 (76)	615 (86)			

* *Χ*^2^ test. ^∮^ logistic regression; ^‖^ reference level. Abbreviations: OR—odds ratio; CI—confidence interval.

## Data Availability

The raw data supporting the conclusions of this article are available upon request.
